# Shyness and Adjustment in Early Childhood in Southeast China: The Moderating Role of Conflict Resolution Skills

**DOI:** 10.3389/fpsyg.2021.644652

**Published:** 2021-04-01

**Authors:** Jingjing Zhu, Rui Fu, Yan Li, Min Wu, Tingting Yang

**Affiliations:** ^1^Department of Preschool Education, Shanghai Normal University, Shanghai, China; ^2^Department of Pediatrics, Center for Violence Prevention, Children's Hospital of Philadelphia, Philadelphia, PA, United States

**Keywords:** shyness, conflict resolution skills, adjustment, preschoolers, China

## Abstract

The massive social change in urban China today has led to a decline in the adaptive implications of shyness for child adjustment, yet evidence of this trend in young children is limited. Moreover, the underlying mechanisms that help to explain the associations between shyness and maladjustment remains poorly understood. The primary goal of the present study was to explore the moderating role of conflict resolution skills in the links between shyness and socio-emotional and school adjustment among urban Chinese preschoolers. Data were collected from 360 children (44.4% girls, *M*_age_ = 4.72 years, *SD* = 0.63) in kindergartens using parent ratings, teacher ratings, and child interviews. The analyses indicated that the relations between shyness and adjustment were moderated by child conflict resolution skills, which served to buffer shy children from adjustment problems. The results were discussed in terms of the implications of conflict resolution skills for early adjustment of shy preschoolers in the Chinese context.

## Introduction

Shyness is a temperamental trait characterized by overt indices of fear and anxiety in challenging social settings (Rubin et al., 2009). It is often manifested in wary, vigilant, and sensitive behavior toward social novelty and/or perceived social evaluation. In the classic conceptual model proposed by Asendorpf (1990), shyness is a form of social withdrawal alongside the other two forms, namely, unsociability and social avoidance. From the perspective of approach and avoidance motivations in social settings, shyness has been suggested to differ from the other forms of social withdrawal as it originates from a combination of high approach–avoidance motivations. Shy children desire to interact with others (i.e., high social approach), but at the same time, their approach tendency is impeded by an elevated anxiety and lack of self-confidence in face of social-evaluation situations (i.e., high social avoidance) (e.g., Asendorpf, 1990; Coplan and Armer, 2007). In contrast, unsociability and social avoidance jointly derive from a low approach motivation for social interaction. From a behavioral perspective, shyness is commonly shown as wary and anxious reactions in social situations rather than as an explicit display of a strong desire for solitude, which is an important aspect of other forms of social withdrawal (Asendorpf, 1993; Chen, 2015).

Behavioral and emotional reactivity to novel objects or circumstances in early childhood is believed to be a major developmental antecedent of shyness (Fox et al., 2005; Stevenson-Hinde et al., 2011). As this antecedent entails a biologically based behavioral tendency, shyness that emerges in early childhood is believed to be a relatively stable trait across developmental periods (Kagan et al., 1986). Despite less research has been conducted in young children as compared to adolescents, anxiety-charged shyness in early childhood has been shown to be a risk factor for a host of social, emotional, and school adjustment difficulties (e.g., Rubin et al., 2009; Hipson et al., 2019; Zhu et al., 2019).

Nevertheless, not all shy young children experience social and emotional maladjustment to the same extent. It has been postulated that individual characteristics such as problem-solving styles may moderate the negative impacts of negative social behavior and experiences on socioemotional well-being (e.g., Kingsbury et al., 2013; Wang et al., 2020). However, there is little research on the moderating role of resolution skills during peer conflicts in young children, particularly in early childhood that marks the beginning of socioemotional development (Coplan and Ooi, 2014). Moreover, the buffering or protective function of conflict resolution skills may be particularly important in group-oriented societies, such as Chinese society, where the maintenance of group harmony and interpersonal relationships are highly emphasized (e.g., Chen and French, 2008). As a result, the significance of conflict resolution skills in protecting shy children from social and emotional problems may be pronounced. The primary purpose of the present study was to explore the buffering role of conflict resolution skills in the associations between shyness and socio-emotional and school adjustment difficulties in Chinese preschoolers.

## Shyness in Early Childhood

The preschool setting is regarded as stressful and challenging for children because of the presence of unfamiliar peers and social-evaluative concerns in peer interaction (Rudasill and Kalutskaya, 2014; Coplan et al., 2018). Research has shown individual differences in reactions to this setting; whereas some young children are relaxed and display a low level of distress, others tend to be anxious and fearful (Burgess et al., 2006; Markovic et al., 2013). Early childhood shyness remains stable across development periods and is evidenced to increase with age largely due to the continuation of temperament impacting one's social experiences in later years (Asendorpf, 1991; Fox et al., 2001; Rubin et al., 2009). In support of the abovementioned motivational underpinning of shyness, Coplan et al. (2004) and Coplan and Armer (2007) provided some of the first evidence that during free play with peers, shy preschoolers tend to exhibit reticence (e.g., watching other children without joining in) and parallel play and that this display of shyness was noticeable to and negatively perceived among preschoolers. Recent scholarship has shown that shyness in early childhood is concurrently and longitudinally associated with peer difficulties in kindergarten and elementary school (Nelson et al., 2005; Gazelle and Spangler, 2007), school avoidance and poor academic performance during adolescence (Buhs et al., 2015; Sette et al., 2017), and social anxiety disorders and depression during adolescence and into adulthood (Gladstone and Parker, 2006; Poole et al., 2017).

The psychological significance attributed to any social behavior is largely a function of the social context within which it is produced (Rubin et al., 2009). This social context is composed of children's daily routines and social activities with significant others (mainly parents, teachers, and peers), and these social interactions are, in large part, culturally determined and defined (Chen and French, 2008). According to the contextual–developmental perspective proposed by Chen (2012), cultural beliefs and values provide a reference frame for social evaluations of and responses to children's shyness and its developmental implications. Adults and peers may evaluate and respond to shy behavior in alignment with cultural values in the society. Whereas, positive evaluations and responses may lead to optimal adjustment outcomes among shy children, negative feedback is likely linked with unfavorable development. As such, in Western individualistic societies where social initiative and self-expression are highly valued as a means for achieving independence and assertiveness, shy children are at an enhanced risk for maladjustment in multiple domain (Chen, 2019; see Rubin et al., 2009 for review). In contrast, in group-oriented societies where the maintenance of relationship harmony and interpersonal relatedness are emphasized as an optimal social goal, shy children's behavioral restraint and wariness are regarded as indicators of social maturity. In the traditional Chinese society, parents tend to interpret shy behavior as compliance and respectfulness (Chen et al., 1998). This argument is well-supported by other early works of Chen and colleagues that show that shyness was positively associated with social competence, academic achievement, and psychological well-being (e.g., Chen et al., 1992, 1995).

Interestingly, there has been emerging research that the changing economic and political climate in China is being accompanied by an increasing cultural preference for more assertive, expressive, and initiative-taking behaviors in social interaction (e.g., Wang and Huang-pu, 2007; Chen et al., 2019), which is particularly pronounced in urban areas of China. As a result, the adaptive meaning of shyness has been declining among urban Chinese children and adolescents (Chen, 2019), which is revealed in negative parental attitudes toward child shyness. For example, urban shy children were perceived as socially incompetent by parents and teachers and received less parental support (Chen et al., 2014; Liu et al., 2015; Li et al., 2016a). With respect to the implications of shyness, recent studies suggest that similar to previous findings in North American societies, it is associated with loneliness, depression, peer exclusion and victimization, and poor academic achievement (Chen et al., 2011; Liu et al., 2014; Coplan et al., 2017). Nonetheless, it should be noted that these studies were conducted mostly in samples of Chinese preadolescents and adolescents. It remains largely uncertain the extent to which the contemporary emphasis on new behavioral qualities in urban China has impacted preschoolers, given that they are relatively less prone to social influence than school-age children (e.g., Nelson et al., 2012; Li et al., 2016b). In the current study, we aimed to examine this issue in order to extend the relatively limited literature in early childhood.

## Conflict Resolution Skills in Urban Chinese Context

The majority of young children participate in frequent yet short-lived conflicts with peers (Chen et al., 2001). These conflicts often involve the distribution of tangible resources (e.g., toys), and throughout the preschool and kindergarten years, issues of conflict become more mental and social, such as over different play ideas (Chen et al., 2001; Pieng and Okamoto, 2020). When involved in peer conflicts, young children exhibit different resolution strategies, and according to the Dual Concern Model (Pruitt and Carnevale, 1993), there are mainly three types of strategies, namely, solution-orientation, control, and non-confrontation, depending on the level of a child's concerns for oneself vs. others in a conflict (Thayer et al., 2008; Gao et al., 2017; Wang et al., 2020). Specifically, solution-orientation strategies are constructive and adaptive strategies as they coordinate target child's and opponent's viewpoints and needs, indicating an advanced level of social understanding and perspective taking. Control strategies are the ones children use to take control of the conflictual interaction for their own interest, reflecting high concern for oneself and low concern for others. Lastly, non-confrontational strategies include avoidant and withdrawal from a disagreement and they are driven by low concern for both oneself and others.

Cross-cultural theory and empirical research has suggested that the implications of different conflict resolution strategies are largely culturally determined (e.g., Thayer et al., 2008; Gao et al., 2017). In the Chinese society where the adaptive meaning of conflict resolution strategies in children is scant, solution-oriented strategies have been associated with friendship satisfaction (Gao et al., 2017) and fewer psychological problems among victimized children (Wang et al., 2020) whereas opposite associations hold for control strategies. Consistent with the existing literature in Western societies (e.g., Mayeux and Cillessen, 2003; Marceau et al., 2015), these findings suggest solution-oriented strategies as an indicator of high conflict resolution skills, while control strategies, a lack of conflict resolution skills. In comparison, less research on the function of non-confrontational strategies has been conducted in urban Chinese children, and in one of the only studies (Wang et al., 2020), avoiding and withdrawing from a conflict is regarded as an indication of social incompetence and has become maladaptive in the urban context. Because how young children interpret and respond to peer conflict may shape both their style of interaction with peers and their behavioral reputation in later developmental periods (Mayeux and Cillessen, 2003), it would be interesting to examine the links between conflict resolution skills and adjustment among urban Chinese preschoolers.

## Moderating Role of Conflict Resolution Skills on Relations Between Shyness and Adjustment

Literature has suggested that individual and social-contextual factors may mediate the associations between shyness and adjustment (e.g., Greco and Morris, 2005; Chen and French, 2008). For example, Gao et al. (2020) reported that shy young adults in China showed low levels of interpersonal competence, which in turn increased their likelihood of depression. Similarly, in two studies of Chinese adolescents, indicators of peer relationship quality (i.e., peer rejection, victimization, and peer preference) mediated the links between shyness and internalizing problems and school maladjustment (Coplan et al., 2017; Liu et al., 2019). Relatively, another mechanism where individual and social–contextual factors may function to moderate the shyness-adjustment link is less explored. Existing studies in urban China have suggested that being academically competent and having the skills to effectively communicate with peers may be important protective factors for shy children (Yang et al., 2014; Zhu et al., 2019). To expand research in this area, we attempted to explore the moderating effects of children's conflict resolution skills on relations between shyness and adjustment problems. We believe that this study may help us understand the processes involved in the development of shyness and inform prevention and intervention efforts that aim at addressing shy children's vulnerabilities from the early years.

As argued earlier, shy young children in urban China are likely to have emotional and relationship difficulties because their anxious and restraint behaviors are perceived as socially incompetent and incompatible with an increasing emphasis on assertiveness and initiative-taking in the society. The adjustment problems that these shy children encounter may be particularly evident for those who have low conflict resolutions skills because of their heightened emotional arousal in stressful peer interaction. Relative to non-shy children, shy children tend to use more internalizing strategies that demand little self-assertiveness in peer interactions (Findlay et al., 2009; Walker and Henderson, 2012; Walker et al., 2014). When employing hostile, avoidant, or other negative behaviors in face of conflicts, shy children are more susceptible to peer rejection and exclusion, which may in turn make them internalize such relationship stress (e.g., Prakash and Coplan, 2003; Findlay et al., 2009). On the contrary, high levels of conflict resolution skills may serve to protect shy young children from social and emotional problems. Despite their anxious and restraint behaviors in the presence of social evaluations, shy children when showing constructive resolution behaviors in resource-limited situations are likely to be perceived as well-regulated. This behavioral demonstration of self-control is highly encouraged as in Chinese society, human behavior is believed to be malleable and controllable (Stevenson et al., 1990; Chen and French, 2008). As a result, conflict resolution competence may be advantageous to enhance shy preschoolers' peer relationship quality, which may lower their risk for social and emotional maladjustment. These arguments are supported by Kingsbury et al. (2013)'s finding that compared with other shy children who relied on internalizing strategies in response to peer conflicts, those who applied solution-oriented strategies reported higher social satisfaction. Similarly, one recent study conducted in urban Chinese adolescents suggests that solution-oriented strategies employed in peer conflict, indicating strong conflict resolution competence, buffer against the psychological difficulties of victimized youth (Wang et al., 2020).

## The Present Study

The purpose of the present study was to examine the role of conflict resolution skills in moderating the links between shyness and social, emotional, and school adjustment among urban Chinese preschoolers. A sample of preschool-age children participated in the study. We assessed children's conflict resolution skills through interviewing the children and collected data on children's shyness from mother reports. In addition, we collected teacher-report data on children's social, emotional, and school adjustment in a follow-up study 18 months later.

Based on the literature (e.g., Chen et al., 2011; Liu et al., 2014) and our previous discussion, we hypothesized that shyness would be positively associated with indices of maladjustment (e.g., asocial behavior, peer exclusion, anxiety, and learning problem) in urban Chinese preschoolers. We also hypothesized that these associations would be moderated by the levels of preschoolers' conflict resolution skills. In particular, the positive associations between shyness and maladjustment would be attenuated by high levels of conflict resolution skills.

## Methods

### Participants

The participants in the study consisted of 360 young children (200 boys, *M*_age_ = 4.72 years, *SD* = 0.63) in 13 classes in three kindergartens in a metropolitan city in Southeast China, with ~25–30 children in each class. Chinese children typically enroll in a kindergarten for 3 years (i.e., at ages 3–4 years attending junior class, 4–5 years attending middle class, and 5–6 years attending senior class). Among the participants, 195 children are from junior class (age range = 3.58–5.41 years, *M*_age_ = 4.28, *SD* = 0.31) and 165 children are from middle class (age range = 4.83–6.42 years, *M*_age_ = 5.26, *SD* = 0.29). All participants in the present study were from middle-class families (approximately 13,000 Chinese yuan or US$2,000 for monthly incomes). Of the mothers and fathers of participating young children, approximately 14.5% and 15.0% had completed high school, 68.7% and 77.4% had a bachelor's degree, and 16.8% and 7.6% had a postgraduate degree. The demographic data for the sample were comparable to the census data of Shanghai released by Bureau of Statistics of China (2010). All children in the present study were of Han nationality, which is the predominant ethnic group (nearly 97% of the population) in China.

### Measures

#### Mother Reports of Shyness

At Time 1, mothers completed the Chinese version of Child Social Preference Scale (CSPS; Coplan et al., 2004; Li et al., 2016b). The CSPS has good psychometric properties and is evidenced to be valid in early childhood in both Western (Coplan et al., 2004) and non-Western cultural contexts (Li et al., 2016b). Of particular interest for the present study was the shyness subscale, which included seven items (e.g., “My child seems to want to play with other children, but is sometimes nervous to”). Mothers rated, on a five-point scale, ranging from 1 (*not true at all*) to 5 (*very true*), how well each item described the child. Item scores for this subscale were averaged to form the shyness variable, with higher scores indicating more shyness. We used confirmatory factor analysis (CFA) to assess the construct validity of the shyness subscale. The results showed that the shyness subscale exhibited adequate construct validity, χ^2^ = 50.19, *df* = 14, χ^2^/*df* = 3.59, *p* < 0.001, NFI = 0.95, CFI = 0.96, IFI = 0.96, RMSEA = 0.08. The internal reliability of the measure (Cronbach's α) was 0.87 in the present study.

#### Conflict Resolution Skills

At Time 1, conflict resolution skills were assessed by administering an adapted version of Social Knowledge Interview that includes three conflict vignettes (Asher and Renshaw, 1981). Each vignette was presented with a picture and extensive explanations to ensure participants' understanding of the hypothetical scenario. Next, the research assistant asked each child to put forward solutions to each conflict scenario. Each time after the child proposed a solution, the research assistant asked for additional solutions until the child indicated that s/he had given all solutions to address the conflict described in the vignette. Following the instruction described by Gai (2007) and Asher and Renshaw (1981), each proposed solution was coded as positive (e.g., negotiating, setting a general rule, sharing/turn-taking), neutral (e.g., ask mom and/or dad to buy another toy”), or negative (e.g., hitting others, threatening others, avoiding the conflict). Positive solutions were given a score of 2, neutral solutions were given a score of 1, and negative solutions were given a score of 0. All the scores were aggregated with higher scores, indicating greater child conflict resolution skills. We trained two independent raters to rigorously follow the coding scheme, and the intercoder reliability was computed for 20% of the sample (*n* = 72). The interview data were then coded by the trained raters after the interrater reliability was achieved at 0.90.

#### Teacher Ratings

At Time 2, the head teacher in each class was requested to rate each student in the class on school-related social and emotional problems, using the Chinese version of Child Behavior Scale (CBS; Ladd and Profilet, 1996). The CBS consisted of six subscales, using a three-point scale, ranging from 1 (*doesn't apply*) to 3 (*certainly applies*). For the current study, three subscales were used to assess asocial behavior (six items; e.g., “solitary child,” Cronbach's α = 0.83, good construct validity from results of CFA as shown in χ^2^ = 36.16, *df* = 9, χ^2^/*df* = 4.01, *p* < 0.001, NFI = 0.95, CFI = 0.96, IFI = 0.96, RMSEA = 0.10), peer exclusion (seven items; e.g., “peers refuse to let child play with them,” Cronbach's α = 0.88; good construct validity: χ^2^ = 61.79, *df* = 13, χ^2^/*df* = 4.75, *p* < 0.001, NFI = 0.92, CFI = 0.94, IFI = 0.94, RMSEA = 0.11), and anxiety (four items; e.g., “appears miserable, unhappy, tearful, or distressed,” Cronbach's α = 0.71; this subscale showed good construct validity: χ^2^ = 5.10, *df* = 2, χ^2^/*df* = 2.55, *p* = 0.08, NFI = 0.98, CFI = 0.99, IFI = 0.99, RMSEA = 0.07), and teachers were asked to rate regarding how well each item described the child. According to literature, this scale has satisfactory psychometric properties and has been used often with young children (e.g., Ladd and Profilet, 1996; Booth-Laforce and Oxford, 2008). The teacher-rating scores on each subscale were averaged to form variables of social and emotional adjustment, respectively.

In addition, teachers completed the subscale of learning problems of the Chinese version of Teacher Behavior Rating Scale (TBRS; Nelson et al., 2012). This subscale of four items tapped children's difficulties in academic performance (e.g., “has difficulty in learning”; this subscale showed good construct validity: χ^2^ = 0.08, *df* = 2, χ^2^/*df* = 0.04, *p* = 0.96, NFI = 1.00, CFI = 1.00, IFI = 1.00, RMSEA = 0.00). Teachers rated, on a three-point scale, ranging from 0 (*doesn't apply*) to 2 (*certainly applies*), how well each item described the child. The item scores were averaged for each child, with higher scores indicating greater learning problems. The internal reliability of the measure (Cronbach's α) was 0.83 in the present study.

#### Procedure

All students in the kindergartens were invited to participate in the study, and the participation rate was approximately 98%. Written consent was obtained from parents of the participants. Data were collected at two time points with an 18-month interval. At Time 1 (June of 2015), mothers were requested to rate their children's shyness, and conflict resolution skills were assessed by administering an adapted version of Social Knowledge Interview that includes three conflict vignettes (Asher and Renshaw, 1981). Children were assured that there were no right or wrong answers. At Time 2 (December of 2016), indices of children's social, emotional, and school adjustment were assessed using teacher ratings. The administration of the interviews and measures in the present study was conducted by a group of faculty, who were a part of the research team, and graduate students, who were research assistants, in psychology in China. All the research assistants had received general training in psychology, child development, and school education and specific training on the administration of the interviews and measures in data collection and on coding the interview data for this study. Prior to the distribution of the questionnaires, parents and teachers were assured that their answers would be kept private. Parent questionnaires were sent home by teachers and returned to the preschool themselves upon parent completion. All procedures were in adherence to the approved IRB protocol of this study.

### Analytical Strategy

At Time 2, 65 (18% of the initial sample) children from the original sample graduated and relocated to other cities. There were non-significant differences on the Time 1 variables between children who participated in the follow-up study and those who did not, *t* = −1.01 to 0.078, *p*s > 0.05. Data analysis was conducted using IBM SPSS (version 22.0). First, we examined gender differences on the variables of interest and inter-correlations among these variables. Second, Hayes's PROCESS macro (Hayes, 2018) with non-parametric bootstrapping was conducted to examine the effects of conflict resolution skills in moderating the associations between shyness and child adjustment after controlling for the covariates and the main effects of shyness and conflict resolution skills. Moderations were regarded as significant when a 95% bias-corrected confidence interval (CI) of an interaction term (shyness^*^conflict resolution skills) did not include zero (Preacher and Hayes, 2008). To probe significant interaction terms, simple slope tests using the methods suggested by Aiken and West (1991) were conducted. Then, the Johnson–Neyman (J–N) technique (Johnson and Fay, 1950) was applied to estimate regions of significance for the adjusted effects of shyness on adjustment variables as a function of conflict resolution skills.

## Results

### Descriptive Data

Results from missing data analysis indicated that missingness for all study variables ranged between 3.3 and 18.6%, and Little's MCAR test (Little, 1988) suggested that the data were missing completely at random, χ^2^(14) = 8.86, *p* > 0.05. Results from *t*-tests indicated significant gender differences on conflict resolution skills, peer exclusion, and learning problems, *t* = −2.23, 2.24, and 2.85, *p*s < 0.05. Specifically, girls (*M* = 6.60, *SD* = 2.92) showed higher conflict resolution skills compared to boys (*M* = 5.89, *SD* = 2.71). Boys (*M* = 0.54, *SD* = 0.51) were rated as having more peer exclusion (*M* = 1.27, *SD* = 0.38) and learning problems (*M* = 0.53, *SD* = 0.51) compared to girls (*M* = 1.18, *SD* = 0.32 for peer exclusion; *M* = 0.37, *SD* = 0.46 for learning problems). However, no gender differences were found on mother-rated child shyness (*M* = 2.09 and 2.12, *SD* = 0.73 and 0.76, for boys and girls, respectively; *t* = −0.06, *p* = 0.58), asocial behavior (*M* = 1.31 and 1.23, *SD* = 0.42 and 0.36, for boys and girls, respectively; *t* = 1.23, *p* = 0.22), and anxiety (*M* = 1.27 and 1.29, *SD* = 0.38 and 0.42, for boys and girls, respectively; *t* = −0.75, *p* = 0.45). Child gender was thus included as a covariate in subsequent analyses.

Inter-correlations among all study variables and their means and standard deviations are presented in Table 1. Significant correlations showed that shyness was negatively associated with conflict resolution skills and positively associated with teacher-rated asocial behavior and peer exclusion. Child age was negatively associated with shyness and positively associated with conflict resolution skills, and thus controlled in subsequent analyses.

**Table 1:** Descriptive data and correlations among variables.

**Variables**	**1**	**2**	**3**	**4**	**5**	**6**	**7**
1. Child age	–	−0.16^**^	0.21^***^	−0.05	−0.06	−0.08	−0.04
2. Shyness		–	−0.13^*^	0.28^***^	0.14^**^	0.10^+^	0.04
3. Conflict resolution skills			–	−0.10^+^	0.00	−0.11^+^	0.01
4. Asocial behavior				–	0.67^***^	0.63^***^	0.44^***^
5. Peer exclusion					–	0.46^***^	0.40^***^
6. Anxiety						–	0.34^***^
7. Learning problems							–
*Mean*	4.72	2.10	8.30	1.23	1.27	1.28	0.46
*SD*	0.63	0.75	3.45	0.34	0.39	0.40	0.50

* p < 0.05,

** p < 0.01,

*** *p < 0.001, ^+^p < 0.10*.

### Moderating Effects of Conflict Resolution Skills

The primary goal of the current study was to examine the moderating effects of conflict resolution skills in the relations between shyness and social, emotional, and school adjustment. We tested the effects of shyness and conflict resolution skills (and their interaction) in relation to the adjustment variables (i.e., asocial behavior, peer exclusion, anxiety, and learning problems), while controlling for child gender. Shyness and conflict resolution skills were standardized prior to being entered into the models.

As shown in Table 2, significant interactions were found between shyness and conflict resolution skills in predicting asocial behavior (*β* = −0.14, *B* = −0.15, *SE* = 0.06, *t* = −2.51, *p* < 0.05), peer exclusion (*β* = −0.13, *B* = −0.13, *SE* = 0.06, *t* = −2.11, *p* < 0.05), anxiety (*β* = −0.16, *B* = −0.18, *SE* = 0.06, *t* = −2.94, *p* < 0.01), and learning problems (*β* = −0.13, *B* = 0.13, *SE* = 0.06, *t* = −2.11, *p* < 0.05), respectively, controlling for the effects of gender and age.

**Table 2:** Contributions of shyness, conflict resolution skills, and their interaction in predicting adjustment.

**Adjustment Outcome Predictor**	**B**	**SE**	**t**	**95% CI**
**Asocial behavior**				
Gender	−0.13	0.12	−1.09	[−0.36, 0.10]
Age	0.05	0.11	0.49	[−0.16, 0.26]
Shyness	0.27^***^	0.06	4.67	[0.16, 0.38]
Conflict resolution skills	−0.04	0.06	−0.61	[−0.16, 0.08]
Shyness * Conflict	−0.14^*^	0.06	−2.51	[−0.25, −0.03]
Resolution skills				
**Peer exclusion**				
Gender	−0.30^*^	0.12	−2.37	[−0.54, −0.05]
Age	0.01	0.11	0.10	[−0.21, 0.23]
Shyness	0.14^*^	0.06	2.33	[0.02, 0.26]
Conflict resolution skills	0.07	0.06	1.10	[−0.05, 0.19]
Shyness * Conflict	−0.13^*^	0.06	−2.11	[−0.24, −0.01]
Resolution skills				
**Anxiety**				
Gender	−0.10	0.12	0.88	[−0.13, 0.33]
Age	−0.06	0.10	−0.56	[−0.26, 0.14]
Shyness	0.08	0.06	1.48	[−0.02, 0.19]
Conflict resolution skills	−0.05	0.06	−0.77	[−0.16, 0.07]
Shyness * Conflict	−0.16^**^	0.06	−2.94	[−0.27, −0.05]
Resolution skills				
**Learning problems**				
Gender	−0.31^*^	0.13	−2.47	[−0.56, −0.06]
Age	−0.02	0.11	−0.19	[−0.24, 0.20]
Shyness	0.04	0.06	0.59	[−0.10, 0.16]
Conflict resolution skills	0.03	0.06	0.46	[−0.10, 0.16]
Shyness * Conflict	−0.13^*^	0.06	−2.11	[−0.25, −0.01]
Resolution skills				

*CI, confidence interval*.

* p < 0.05,

** p < 0.01,

*** *p < 0.001*.

**Figure 1:**
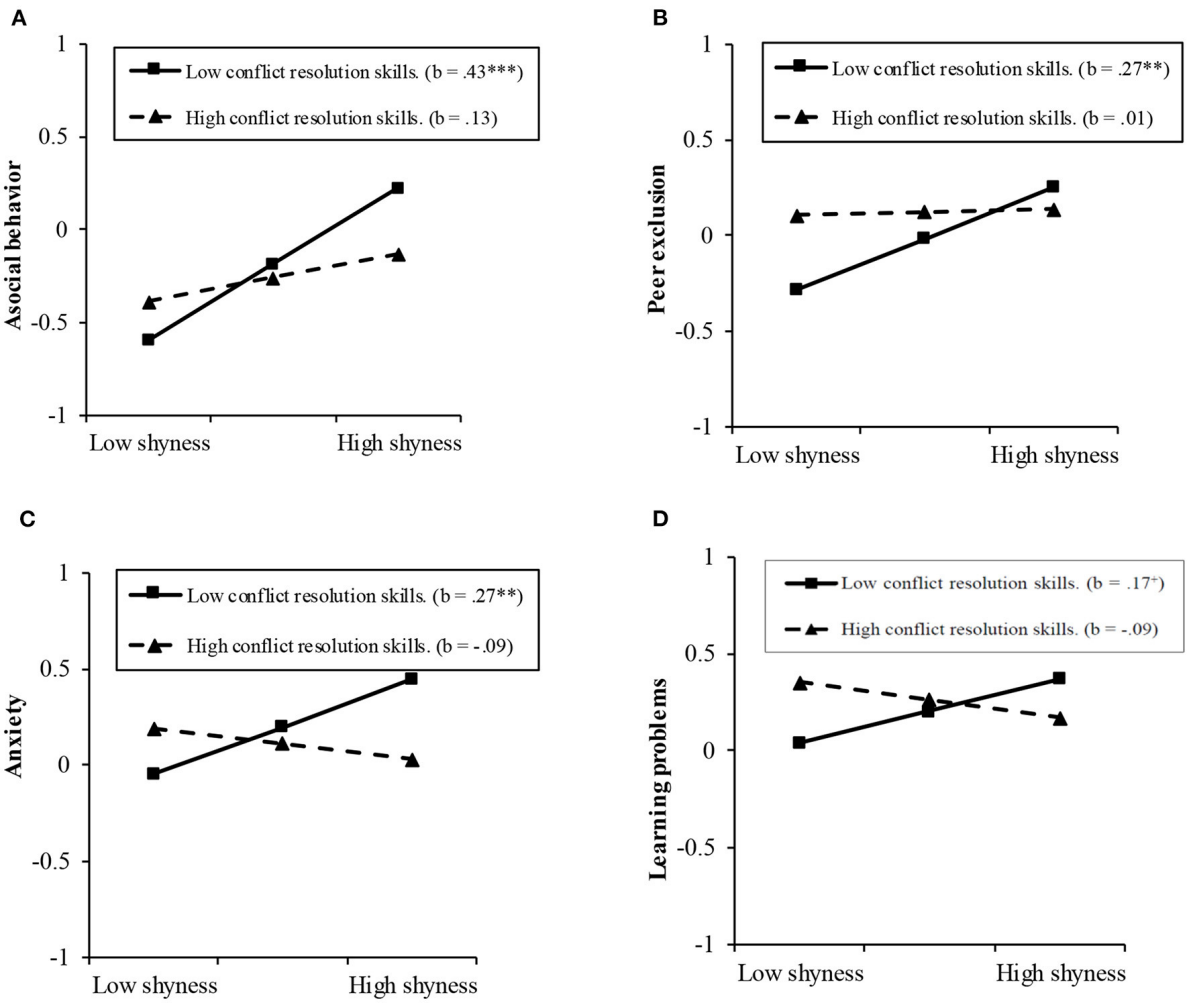
**(A)** Interaction between shyness and conflict resolution skills in predicting asocial behavior. **(B)** Interaction between shyness and conflict resolution skills in predicting peer exclusion. **(C)** Interaction between shyness and conflict resolution skills in predicting anxiety. **(D)** Interaction between shyness and conflict resolution skills in predicting learning problems.

The simple slope effects were examined on Time 1 shyness at high and low values (1 *SD* above and 1 *SD* below the mean) of conflict resolution skills. Results indicated that shyness was positively associated with asocial behavior for children who had low scores on conflict resolution skills, *B* = 0.43, *SE* = 0.08, *t* = 5.14, *p* < 0.001, whereas this association was non-significant for children who had high scores on conflict resolution skills, *B* = 0.13, *SE* = 0.08, *t* = 1.55, *p* > 0.05 (see Figure 1A). In addition, shyness was positively associated with peer exclusion for children who had low scores on conflict resolution skills, *B* = 0.27, *SE* = 0.08, *t* = 3.18, *p* < 0.01, whereas this association was non-significant for children who had high scores on conflict resolution skills, *B* = 0.01, *SE* = 0.09, *t* = 0.18, *p* > 0.05 (see Figure 1B). Similarly, shyness was positively associated with anxiety and learning problems for children who had low scores on conflict resolution skills, *B* = 0.27 and 0.17, *SE* = 0.08 and 0.09, *t* = 3.16 and 1.92, *p* < 0.01 and *p* < 0.10, respectively, whereas these associations were non-significant for children who had high scores on conflict resolution skills, *B* = −0.09 and −0.09, *SE* = 0.08 and 0.09, *t* = −0.99 and −1.05, *p*s > 0.05 (see Figures 1C,D).

**Figure 2:**
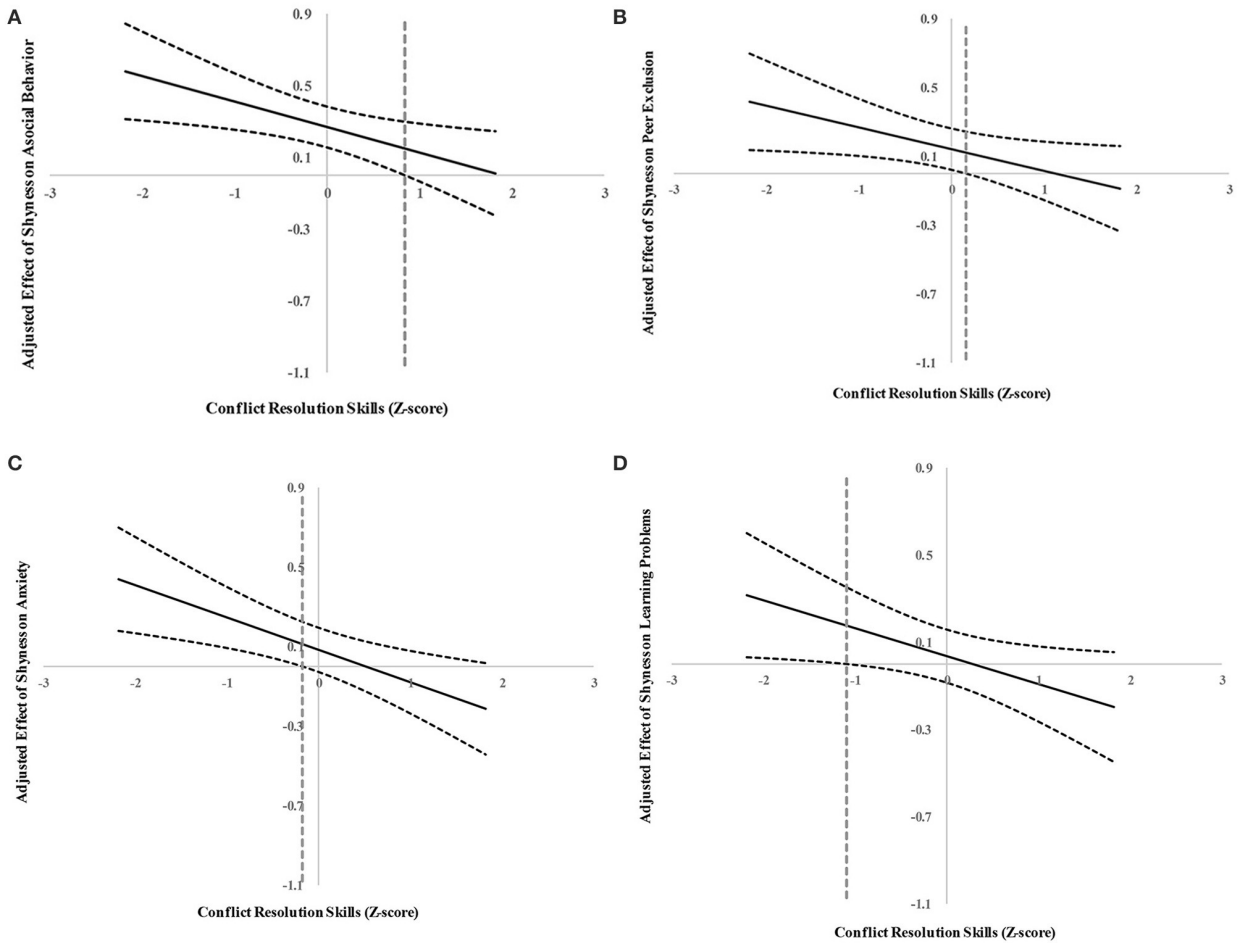
**(A)** Adjusted effect of shyness on asocial behavior using the Johnson–Neyman technique. The dashed vertical line indicates the point along the conflict resolution skills in which shyness regression coefficient transitions from significance (left of the dashed vertical line) to non-statistical significance (right of the dashed vertical line). The value of the dashed vertical line was 0.84. **(B)** Adjusted effect of shyness on peer exclusion using the Johnson–Neyman technique. The dashed vertical line indicates the point along the conflict resolution skills in which shyness regression coefficient transitions from significance (left of the dashed vertical line) to non-statistical significance (right of the dashed vertical line). The value of the dashed vertical line was 0.16. **(C)** Adjusted effect of shyness on anxiety using the Johnson–Neyman technique. The dashed vertical line indicates the point along the conflict resolution skills in which shyness regression coefficient transitions from significance (left of the dashed vertical line) to non-statistical significance (right of the dashed vertical line). The value of the dashed vertical line was −0.18. **(D)** Adjusted effect of shyness on learning problems using the Johnson–Neyman technique. The dashed vertical line indicates the point along the conflict resolution skills in which shyness regression coefficient transitions from significance (left of the dashed vertical line) to non-statistical significance (right of the dashed vertical line). The value of the dashed vertical line was −1.09.

Results of the J–N technique showed that shyness was positively associated with asocial behavior, peer exclusion, anxiety, and learning problems for children with a low level of conflict resolution skills; the associations were non-significant for children with a high level of conflict resolution skills. Specifically, for children who scored lower than 0.84 *SD* and 0.16 *SD* above the average of conflict resolution skills, respectively, the adjusted effects of shyness on asocial behavior and peer exclusion were significantly positive (see Figures 2A,B). When the values were above 0.84 *SD* and 0.16 *SD* of the average conflict resolution skills, respectively, shyness was no longer associated with asocial behavior and peer exclusion. Similarly, for anxiety and learning problems, for children who scored lower than 0.18 *SD* and 1.09 *SD* below the average of conflict resolution skills, respectively, the adjusted effects of shyness on anxiety and learning problems were significantly positive (see Figures 2C,D). When the values were above −0.18 and −1.09 *SD* of the average of conflict resolution skills, respectively, shyness was no longer associated with anxiety and learning problems. In addition, we examined the interaction effects of shyness and gender, conflict resolution skills and gender, and a three-way interaction (shyness and gender and conflict resolution skills) on the adjustment variables and none of the interactions were significant, *B* = −0.19 to 0.17, *SE* = 0.09 to 0.13, *t* = −1.05 to 1.34, *p*s > 0.05.

## Discussion

A growing number of recent studies have shown the implications of shyness for a range of adjustment difficulties in urban Chinese children in middle childhood and beyond, suggesting that behavioral qualities relating to shyness are no longer highly valued in the urban areas of China (Liu et al., 2014, 2015). The current study adds to the literature that shyness was positively associated with social, emotional, and school maladjustment in urban Chinese preschoolers, particularly among those with poor conflict resolution skills. However, the links between shyness and maladjustment were not significant for those preschoolers who had strong conflict resolution skills. These results suggest that in urban settings, the negative implications of shyness for child adjustment may be evident as early as in early childhood and that strong conflict resolution skills may buffer maladjustment in shy young children.

### Shyness Among Preschoolers in Urban China

Our study adds to the literature in the recent decade that among urban Chinese children in early childhood, the adaptive meaning of shyness for child adjustment is in decline (Chen et al., 2005, 2009; Liu et al., 2015; Li et al., 2016b). This is largely attributed to the rapid social and economic changes in contemporary urban China, which might have reshaped how the behavioral qualities of shy children are viewed. The exhibition of anxiety and wariness in shy young preschoolers appears to be perceived as an indicator of social incompetence or even social disinterest by teachers, which are consistent with findings in urban adolescents in China (Chen et al., 2014; Liu et al., 2015). Thus, this finding adds to the literature that as early as in early childhood, behavioral characteristics concerning shyness may no longer contribute positively to child adjustment in the urban settings.

### Shyness and Adjustment: The Moderating Role of Conflict Resolution Skills

There has been consistent evidence that poor conflict resolution skills may result in children responding to conflictual interactions in either an externalizing or internalizing manner (e.g., Killen and de Waal, 2000; Kingsbury et al., 2013). In the study, we found that conflict resolution skills served to mitigate social, psychological, and school problems in shy young children in urban China. This extends the current literature that has mostly focused on the direct effect of conflict resolution competence, by providing first evidence that conflict resolution skills may present as an important protective factor for shy young children in urban China.

Although shy children tend to be anxious and wary about social evaluative situations, their constructive, solution-oriented strategies when in conflict with peers are likely to be perceived as being prosocial and well-behaved, which are highly encouraged in Chinese schools (Chen and French, 2008). As such, these shy preschoolers are likely to establish close relationships with teachers, which would prevent them from having academic difficulties (Hamre and Pianta, 2001; McCormick and O'Connor, 2015). Also, positive relationships with peers and teachers may lower their tendency in concentrating on anxiety and worry in response to potential social interactions at school (e.g., group assignments), but rather motivate them to focus on improving school performance. On the other hand, shy preschoolers' conflict resolution incompetence is more prone to social and emotional difficulties. As posited in Chen's contextual–developmental perspective (Chen, 2012), negative social evaluations and responses constitute an environment that facilitates the development of psychological problems in shy children. Perhaps, when handling peer conflicts passively by worrying and self-blaming (Burgess et al., 2006; Findlay et al., 2009), these shy young children are likely to be perceived as showing insincerity of solving problems by others, which may give rise to peer exclusions or other social problems. Meanwhile, these rejection experiences may reinforce shy young children's focus on the negative emotional components of social stressors and further heighten their tendency to withdraw themselves from peer interactions. Interestingly, we found supporting evidence to the limited literature (Wang et al., 2020) that the use of non-confrontational strategies (coded as negative solutions in this study) has indeed negative influence on children's and adolescents' well-being in the urban settings, particularly those who have already encountered peer problems.

Although empirical studies have shown that shy boys may be more susceptible to maladjustment relative to shy girls (e.g., Coplan et al., 2012; Liu et al., 2015) in China, our findings did not support it. In addition, the main effects of conflict resolution competence on maladjustment and its moderating effects on the link between shyness and maladjustment were similar across gender. Perhaps in urban China, boys and girls are expected to show less shyness yet more self-assertiveness to the same extent as the declining value of shyness appears to be similar for both. Also, conflict resolution skills may be regarded as a desirable competence for both genders. In sum, these findings may reveal the societal trend toward gender egalitarianism in urban China today, which nevertheless needs to be further examined in future studies.

## Limitations and Conclusions

Several limitations of the study should be noted. First, although the present study was a short-term longitudinal study, teacher-report child behavior and adjustment were not administrated at Time 1 because the measures of teacher ratings are typically used among children of age 5 and above and the participants were younger than that at Time 1. Consequently, the stabilities of the adjustment outcomes were not controlled in the analyses. Therefore, one needs to be careful in interpreting the results in terms of causality. Second, the present study solely relied on maternal and teacher reports. Although this approach collected information from two typical socialization settings for preschoolers (i.e., at home and at school), future researchers should consider measuring preschoolers' shyness and adjustment outcomes using other methods, including observation data, child interview, and peer evaluation with age-appropriate rating items. Third, the present study was conducted in Shanghai, the largest city in China, and one needs to be careful in generalizing the present results to less-developed urban or rural regions in the country. There are substantial differences in the scale of social changes across urban regions in the country, which is likely to impact the adaptive meaning of shyness as shown in its implications for preschoolers' maladjustment. Also, the associations between shyness and child maladjustment might be less strong or non-significant in rural areas as literature has shown that shy, wary, and sensitive behaviors may still be viewed as a positive behavioral trait that reflects social maturity, mastery, and understanding rural Chinese youths, despite the fact that they are no longer valued or encouraged in developed areas in China (Chen et al., 2009, 2011). Our findings inform early intervention efforts for shy preschoolers in urban China the value of promoting their conflict resolution strength in attenuating the negative implications of shyness. Specifically, for shy young children, intervention programs should consider focusing on enhancing these children's skills in solving peer conflicts. This extends the findings of one promising early intervention program, *Social Skills Training Facilitated Play Program* (Li et al., 2016c), that training shy preschoolers on solution-oriented strategies in conflict situation appears to be one critical aspect of social skills. Early education teachers can help shy preschoolers better identify and understand their own and other peers' goals and intentions, which have been suggested as a robust predictor of young children's use of solution-oriented strategies (Pieng and Okamoto, 2020).

Taken together, the results of the present study reveal that shyness in early childhood may contribute to social, emotional, and school maladjustment in urban Chinese children and that conflict resolution skills appear to buffer shy preschoolers from social, emotional, and school problems.

## Data Availability Statement

The datasets used and analyzed during the current study will be made available by the corresponding author upon reasonable request.

## Ethics Statement

The studies involving human participants were reviewed and approved by Institutional Review Board of Shanghai Normal University. Written informed consent to participate in this study was provided by the participants' legal guardian/next of kin.

## Author Contributions

JZ drafted the manuscript with support from RF, who also verified the analytical methods. YL designed and supervised the project. MW assisted JZ with data analysis and TY helped with survey administration and data management. All authors contributed to the completion of the final manuscript.

## Conflict of Interest

The authors declare that the research was conducted in the absence of any commercial or financial relationships that could be construed as a potential conflict of interest.

## References

[B1] Aiken L. S. West S. G. (1991). Multiple Regression: Testing and Interpreting Interactions. Newbury Park, CA: Sage.

[B2] Asendorpf J. B. (1990). Beyond social withdrawal: shyness, unsociability, and peer avoidance. Hum. Dev. 33, 250–259. 10.1159/000276522

[B3] Asendorpf J. B. (1991). Development of inhibited children's coping with unfamiliarity. Child Dev. 62, 1460–1474. 10.2307/1130819 1786728

[B4] Asendorpf J. B. (1993). Abnormal shyness in children. J. Child Psychol. Psychiatry 34, 1069–1083. 10.1111/j.1469-7610.1993.tb01774.x 8245133

[B5] Asher S. R. Renshaw P. D. (1981). “Children without friends: social knowledge and social skill training,” in The Development of Children's Friendships, eds Asher S. R. Gottman J. M. (Cambridge: Cambridge University Press), 273–296.

[B6] Booth-Laforce C. Oxford M. L. (2008). Trajectories of social withdrawal from grades 1 to 6: prediction from early parenting, attachment, and temperament. Dev. Psychol. 44, 1298–1313. 10.1037/a0012954 18793064 PMC4305444

[B7] Buhs E. S. Rudasill K. M. Kalutskaya I. N. Griese E. R. (2015). Shyness and engagement: contributions of peer rejection and teacher sensitivity. Early Child. Res. Q. 30(Pt. A), 12–19. 10.1016/j.ecresq.2014.07.010

[B8] Bureau of Statistics of China (2010). 2010 Shanghai Statistical Yearbook. Beijing: China Statistics Press.

[B9] Burgess K. B. Bowker J. C. Rubin K. H. Rose-Krasnor L. Booth-LaForce C. (2006). Social information processing and coping strategies of shy/withdrawn and aggressive children: does friendship matter? Child Dev. 77, 371–383. 10.1111/j.1467-8624.2006.00876.x 16611178 PMC3800105

[B10] Chen D. W. Fein G. G. Killen M. Tam H. (2001). Peer conflicts of preschool children: issues, resolution, incidence, and age-related patterns. Early Educ. Dev. 12, 523–544. 10.1207/s15566935eed1204_3

[B11] Chen X. (2012). Culture, peer interaction, and socioemotional development. Child Dev. Perspect. 6, 27–34. 10.1111/j.1750-8606.2011.00187.x

[B12] Chen X. (2015). Culture, types of social withdrawal, and children's beliefs: an integrative perspective. Br. J. Dev. Psychol. 33, 174–176. 10.1111/bjdp.12086 25765102

[B13] Chen X. (2019). Culture and shyness in childhood and adolescence. New Ideas Psychol. 53, 58–66. 10.1016/j.newideapsych.2018.04.007

[B14] Chen X. Cen G. Li D. He Y. (2005). Social functioning and adjustment in Chinese children: the imprint of historical time. Child Dev. 76, 182–195. 10.1111/j.1467-8624.2005.00838.x 15693766

[B15] Chen X. French D. C. (2008). Children's social competence in cultural context. Annu. Rev. Psychol. 59, 591–616. 10.1146/annurev.psych.59.103006.093606 18154504

[B16] Chen X. Fu R. Li D. Liu J. (2019). Developmental trajectories of shyness-sensitivity from middle childhood to early adolescence in China: Contributions of peer preference and mutual friendship. J. Abnorm. Child Psychol. 1–13. 10.1007/s10802-018-00507-0 30637554

[B17] Chen X. Hastings P. D. Rubin K. H. Chen H. Cen G. Stewart S. L. (1998). Child-rearing attitudes and behavioral inhibition in Chinese and Canadian toddlers: a cross-cultural study. Dev. Psychol. 34, 677–686. 10.1037/0012-1649.34.4.677 9681259

[B18] Chen X. Rubin K. H. Li B. (1995). Social and school adjustment of shy and aggressive children in China. Dev. Psychopathol. 7, 337–349. 10.1017/S0954579400006544

[B19] Chen X. Rubin K. H. Sun Y. (1992). Social reputation and peer relationships in Chinese and Canadian children: a cross-cultural study. Child Dev. 63, 1336–1343. 10.2307/1131559

[B20] Chen X. Wang L. Cao R. (2011). Shyness sensitivity and unsociability in rural Chinese children: relations with social, school, and psychological adjustment. Child Dev. 82, 1531–1543. 10.1111/j.1467-8624.2011.01616.x 21790539

[B21] Chen X. Wang L. Wang Z. (2009). Shyness-sensitivity and social, school, and psychological adjustment in rural migrant and urban children in China. Child Dev. 80, 1499–1513. 10.1111/j.1467-8624.2009.01347.x 19765014

[B22] Chen X. Zhang G. Liang Z. Zhao S. Way N. Yoshikawa H.. (2014). Relations of behavioural inhibition with shyness and social competence in Chinese children: moderating effects of maternal parenting. Infant Child Dev. 23, 343–352. 10.1002/icd.1852

[B23] Coplan R. J. Armer M. (2007). A “multitude” of solitude: a closer look at social withdrawal and nonsocial play in early childhood. Child Dev. Perspect. 1, 26–32. 10.1111/j.1750-8606.2007.00006.x

[B24] Coplan R. J. Liu J. Cao J. Chen X. Li D. (2017). Shyness and school adjustment in Chinese children: the roles of teachers and peers. Sch. Psychol. Q. 32, 131–142. 10.1037/spq0000179 27736120

[B25] Coplan R. J. Ooi L. (2014). “The causes and consequences of “playing alone” in childhood,” in A Handbook of Solitude: Psychological Perspectives on Social Isolation, Social Withdrawal, and Being Alone, eds Coplan R. J. Bowker J. (Hoboken, NJ: Wiley-Blackwell), 111–128.

[B26] Coplan R. J. Ooi L. L. Xiao B. Rose-Krasnor L. (2018). Assessment and implications of social withdrawal in early childhood: a first look at social avoidance. Soc. Dev. 27, 125–139. 10.1111/sode.12258

[B27] Coplan R. J. Prakash K. O'Neil K. Armer M. (2004). Do you “want” to play? Distinguishing between conflicted shyness and social disinterest in early childhood. Dev. Psychol. 40, 244–258. 10.1037/0012-1649.40.2.244 14979764

[B28] Coplan R. J. Zheng S. Weeks M. Chen X. (2012). Young children's perceptions of social withdrawal in China and Canada. Early Child Dev. Care 182, 591–607. 10.1080/03004430.2011.56632825877376

[B29] Findlay L. Coplan R. J. Bowker A. (2009). Keeping it all inside: shyness, internalizing coping strategies and socio-emotional adjustment in middle childhood. Int. J. Behav. Dev. 33, 47–54. 10.1177/0165025408098017

[B30] Fox N. A. Henderson H. A. Marshall P. J. Nichols K. E. Ghera M. M. (2005). Behavioral inhibition: linking biology and behavior within a developmental framework. Annu. Rev. Psychol. 56, 235–262. 10.1146/annurev.psych.55.090902.141532 15709935

[B31] Fox N. A. Henderson H. A. Rubin K. H. Calkins S. D. Schmidt L. A. (2001). Continuity and discontinuity of behavioral inhibition and exuberance: psychophysiological and behavioral influences across the first four years of life. Child Dev. 72, 1–21. 10.1111/1467-8624.00262 11280472

[B32] Gai X. S. (2007). “Development of an emotional and social assessment measure: the SRTB-ES,” in The Research and Practice of Children's School Readiness, eds Gai X. S. (Jilin: Jilin Education Press), 106–116.

[B33] Gao F. Sun Y. Zhou Y. Sang M. Zhao J. Han L. (2020). Shyness and depression: the mediating roles of interpersonal competence, dormitory belonging and inferiority. Child. Youth Serv. Rev. 119:105571. 10.1016/j.childyouth.2020.105571

[B34] Gao Q. Bian R. Liu R. D. He Y. Oei T. P. (2017). Conflict resolution in Chinese adolescents' friendship: links with regulatory focus and friendship satisfaction. J. Psychol. 151, 268–281. 10.1080/00223980.2016.1270887 28075708

[B35] Gazelle H. D. Spangler T. (2007). Early childhood anxious solitude and subsequent peer relationships: maternal and cognitive moderators. J. Appl. Dev. Psychol. 28, 515–535. 10.1016/j.appdev.2007.06.006 18496601 PMC2390866

[B36] Gladstone G. L. Parker G. B. (2006). Is behavioral inhibition a risk factor for depression? J. Affect. Disord. 95, 85–94. 10.1016/j.jad.2006.04.015 16808978

[B37] Greco L. A. Morris T. L. (2005). Factors influencing the link between social anxiety and peer acceptance: contributions of social skills and close friendships during middle childhood. Behav. Ther. 36, 197–205. 10.1016/S0005-7894(05)80068-1

[B38] Hamre B. K. Pianta R. C. (2001). Early teacher–child relationships and the trajectory of children's school outcomes through eighth grade. Child Dev. 72, 625–638. 10.1111/1467-8624.00301 11333089

[B39] Hayes A. F. (2018). Partial, conditional, and moderated mediation: quantification, inference, and interpretation. Commun. Monogr. 85, 4–40. 10.1080/03637751.2017.1352100

[B40] Hipson W. E. Coplan R. J. Séguin D. G. (2019). Active emotion regulation mediates links between shyness and social adjustment in preschool. Soc. Dev. 28, 893–907. 10.1111/sode.12372

[B41] Johnson P. O. Fay L. C. (1950). The Johnson-Neyman technique, its theory and application. Psychometrika 15, 349–367. 10.1007/BF02288864 14797902

[B42] Kagan J. Resnick J. S. Snidman N. (1986). “Temperamental inhibition in early childhood,” The Study of Temperament: Changes, Continuities, and Challenges, eds in Plomin R. Dunn J. (Hillsdale, NJ: Erlbaum), 53–65.

[B43] Killen M. de Waal F. B. M. (2000). “The evolution and development of morality,” in Natural Conflict Resolution, eds Aureli F. de Waal F. B. M. (Berkeley, CA: Berkeley University Press), 352–372.

[B44] Kingsbury M. Coplan R. J. Rose-Krasnor L. (2013). Shy but getting by? An examination of the complex links among shyness, coping, and socioemotional functioning in childhood. Soc. Dev. 22, 126–145. 10.1111/sode.12003

[B45] Ladd G. W. Profilet S. M. (1996). The child behavior scale: a teacher-report measure of young children's aggressive, withdrawn, and prosocial behaviors. Dev. Psychol. 32, 1008–1024. 10.1037/0012-1649.32.6.1008

[B46] Li Y. Coplan R. J. Archbell K. A. Bullock A. Chen L. (2016a). Chinese kindergarten teachers' beliefs about young children's classroom social behavior. Early Child. Res. Q. 36. 122–132. 10.1016/j.ecresq.2015.10.008

[B47] Li Y. Coplan R. J. Wang Y. Yin J. Zhu J. Gao Z.. (2016c). Preliminary evaluation of a Social Skills Training and Facilitated Play early intervention programme for extremely shy young children in China. Infant Child Dev. 25, 1–10. 10.1002/icd.1959

[B48] Li Y. Zhu J. Coplan R. J. Gao Z. Xu P. Li L.. (2016b). Assessment and implications of social withdrawal subtypes in young Chinese children: the Chinese version of the child social preference scale. J. Genet. Psychol. 177, 97–101. 10.1080/00221325.2016.1174100 27177123

[B49] Little R. J. (1988). A test of missing completely at random for multivariate data with missing values. J. Am. Stat. Assoc. 83, 1198–1202. 10.1080/01621459.1988.10478722

[B50] Liu J. Bowker J. C. Coplan R. J. Yang P. Li D. Chen X. (2019). Evaluating links among shyness, peer relations, and internalizing problems in Chinese young adolescents. J. Res. Adolesc. 29, 696–709. 10.1111/jora.12406 29777546

[B51] Liu J. Chen X. Coplan R. J. Ding X. Zarbatany L. Ellis W. (2015). Shyness and unsociability and their relations with adjustment in Chinese and Canadian children. J. Cross Cult. Psychol. 46, 371–386. 10.1177/0022022114567537

[B52] Liu J. Coplan R. J. Chen X. Li D. Ding X. Zhou Y. (2014). Unsociability and shyness in Chinese children: concurrent and predictive relations with indices of adjustment. Soc. Dev. 23, 119–136. 10.1111/sode.12034

[B53] Marceau K. Zahn-Waxler C. Shirtcliff E. A. Schreiber J. E. Hastings P. Klimes-Dougan B. (2015). Adolescents', mothers', and fathers' gendered coping strategies during conflict: youth and parent influences on conflict resolution and psychopathology. Dev. Psychopathol. 27, 1025–1044. 10.1017/S0954579415000668 26439060 PMC4632642

[B54] Markovic A. Rose-Krasnor L. Coplan R. J. (2013). Shy children's coping with a social conflict: the role of personality self-theories. Pers. Individ. Dif. 54, 64–69. 10.1016/j.paid.2012.08.002

[B55] Mayeux L. Cillessen A. N. H. (2003). Development of social problem solving in early childhood: stability, change, and associations with social competence. J. Genet. Psychol. 164, 153–173. 10.1080/00221320309597975 12856813

[B56] McCormick M. P. O'Connor E. E. (2015). Teacher–child relationship quality and academic achievement in elementary school: does gender matter? J. Educ. Psychol. 107, 502–516. 10.1037/a0037457

[B57] Nelson L. J. Hart C. H. Yang C. Wu P. Jin S. (2012). An examination of the behavioral correlates of subtypes of nonsocial play among Chinese preschoolers. Merrill Palmer Q. 58, 77–109. 10.1353/mpq.2012.0006

[B58] Nelson L. J. Rubin K. Fox N. (2005). Social withdrawal, observed peer acceptance, and the development of self-perceptions in children ages 4 to 7 years. Early Educ. Dev. 20, 185–200. 10.1016/j.ecresq.2005.04.007

[B59] Pieng P. Okamoto Y. (2020). Examining preschool children's intention understanding and their conflict resolution strategies. Early Child. Educ. J. 48, 597–606. 10.1007/s10643-020-01020-0

[B60] Poole K. L. Van Lieshout R. J. Schmidt L. A. (2017). Shyness and sociability beyond emerging adulthood: implications for understanding the developmental sequelae of shyness subtypes. J. Soc. Clin. Psychol. 36, 316–334. 10.1521/jscp.2017.36.4.316

[B61] Prakash K. Coplan R. J. (2003). Shy skaters? Shyness, coping and adjustment outcomes in female adolescent figure skaters. Athletic Insight 5, 1–19.

[B62] Preacher K. J. Hayes A. F. (2008). Asymptotic and resampling strategies for assessing and comparing indirect effects in multiple mediator models. Behav. Res. Methods 40, 879–891. 10.3758/BRM.40.3.879 18697684

[B63] Pruitt D. G. Carnevale P. J. (1993). Negotiation in Social Conflict. Pacific Grove, CA: Brooks/Cole.

[B64] Rubin K. H. Coplan R. J. Bowker J. C. (2009). Social withdrawal in childhood. Annu. Rev. Psychol. 60, 141–171. 10.1146/annurev.psych.60.110707.163642 18851686 PMC3800115

[B65] Rudasill K. M. Kalutskaya I. (2014). Being shy at school. Sex Roles 70, 267–273. 10.1007/s11199-014-0345-0

[B66] Sette S. Zava F. Baumgartner E. Baiocco R. Coplan R. J. (2017). Shyness, unsociability, and socio-emotional functioning at preschool: the protective role of peer acceptance. J. Child Fam. Stud. 26, 1196–1205. 10.1007/s10826-016-0638-8

[B67] Stevenson H. W. Chen C. Uttal D. H. (1990). Beliefs and achievement: a study of Black, White, and Hispanic children. Child Dev. 61, 508–523. 10.2307/1131111 2344786

[B68] Stevenson-Hinde J. Shouldice A. Chicot R. (2011). Maternal anxiety, behavioral inhibition, and attachment. Attach. Hum. Dev. 13, 199–215. 10.1080/14616734.2011.562409 21506027

[B69] Thayer S. M. Updegraff K. A. Delgado M. Y. (2008). Conflict resolution in Mexican American adolescents' friendships: links with culture, gender and friendship quality. J. Youth Adolesc. 37, 783–797. 10.1007/s10964-007-9253-8 19183710 PMC2633221

[B70] Walker O. L. Henderson H. A. (2012). Temperament and social problem solving competence in preschool: influences on academic skills in early elementary school. Soc. Dev. 21, 761–779. 10.1111/j.1467-9507.2011.00653.x 23355765 PMC3551269

[B71] Walker O. L. Henderson H. A. Degnan K. A. Penela E. C. Fox N. A. (2014). Associations between behavioral inhibition and children's social problem-solving behavior during social exclusion. Soc. Dev. 23, 487–501. 10.1111/sode.12053 25360063 PMC4209474

[B72] Wang C. Huang-pu X. (2007). A survey and analysis of urban-rural family education. J. Changzhi Univ. 24, 34–36.

[B73] Wang Z. Chen X. Liu J. Bullock A. Li D. Chen X.. (2020). Moderating role of conflict resolution strategies in the links between peer victimization and psychological adjustment among youth. J. Adolesc. 79, 184–192. 10.1016/j.adolescence.2020.01.002 31978837

[B74] Yang F. Chen X. Wang L. (2014). Relations between aggression and adjustment in Chinese children: moderating effects of academic achievement. J. Clin. Child Adolesc. Psychol. 43, 656–669. 10.1080/15374416.2013.782816 23557214

[B75] Zhu J. Li Y. Wood K. R. Coplan R. J. Chen X. (2019). Shyness and socioemotional functioning in young Chinese children: The moderating role of receptive vocabulary. Early Educ. Dev. 30, 590–607. 10.1080/10409289.2019.1572481

